# Effects of physical exercise, LEGO, and Minecraft activities on anxiety in underserved children with autism: Study design and methodological strategies

**DOI:** 10.1016/j.mex.2023.102332

**Published:** 2023-08-18

**Authors:** Angelina Lo, Lea Ann Lowery, Karen Kuhlthau, Robert A. Parker, James Chan, Fadia Haddad, Shlomit Radom-Aizik, Jean-G. Gehricke

**Affiliations:** a University of California, Irvine, School of Medicine. 1001 Health Sciences Rd, Irvine, CA 92617, USA; b Thompson Center for Autism & Neurodevelopmental Disorders, University of Missouri. 205 Portland Street, Columbia, MO 65211, USA; c Massachusetts General Hospital (MGH), Harvard University. 125 Nashua Street 8th Floor Boston, MA 02114, USA; d Pediatric Exercise & Genomics Research Center, Department of Pediatrics, University of California, Irvine. 101 Academy, Suite 150, Irvine, CA 92617, USA; e The Center for Autism & Neurodevelopmental Disorders, Department of Pediatrics, University of California, Irvine, 2500 Red Hill Ave #100, Santa Ana, CA 92705, USA

**Keywords:** Autism, Anxiety, Physical exercise, Play therapy, Lego, Minecraft, Physical Exercise to Reduce Anxiety

## Abstract

Anxiety is a common comorbidity for individuals with ASD, and there is some preliminary data about the efficacy of physical exercise to alleviate anxiety. However, we are not aware of any studies that have compared the effects of a physical exercise program on anxiety in underserved children with ASD using a randomized controlled research design. This paper describes a method to evaluate and compare the efficacy of an 8-week physical exercise intervention with a sedentary play intervention to alleviate anxiety in young children with autism spectrum disorders (ASD) from underserved backgrounds. We assessed anxiety and its physical symptoms using the parent-rated Child Behavior Checklist DSM-5 anxiety (CBCL DSM-5) subscale, the child-rated Screen for Childhood Anxiety Related Emotional Disorder (SCARED), the parent-rated Child's Sleep Habits Questionnaire (CSHQ), and salivary cortisol. We also utilized the Physical Activity Questionnaire for Older Children (PAQ-C) to assess physical activity level and identify compounds. Unique components of this study include:

• Implementation of novel physical exercise and sedentary play interventions that have been designed for children with ASD.
• Recruitment of predominantly underserved and non-English speaking families.

## Graphical abstract

**Figure:**
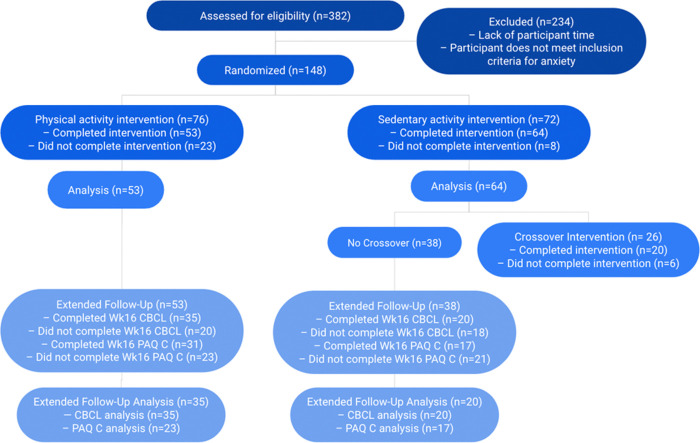

Specifications table

**Table:** 

Subject area:	*Psychology*
More specific subject area:	Anxiety and Autism Spectrum Disorders
Name of your method:	Physical Exercise to Reduce Anxiety
Name and reference of original method:	*NA*
Resource availability:	Sedentary Activity Resources: Minecraft: https://www.minecraft.net/en-us Lego: https://www.lego.com/en-us/ Cortisol Collection & Analysis: Salimetrics Children Swab®: https://salimetrics.com/product/swab-method-children-50pk/ Cortisol Assay: https://salimetrics.com/assay-kit/salivary-cortisol-elisa-kit/ Molecular Devices Microplate Reader: https://www.marshallscientific.com/Molecular-Devices-SpectraMax-384-Plus-Microplate-p/md-sm384.htm SoftMax Pro Software: https://www.moleculardevices.com/products/microplate-readers/acquisition-and-analysis-software/softmax-pro-software

## Method details

### Study background

Anxiety is one of the most frequent comorbidities in children and adolescents with ASD [[1], [2], [3], [4]], which can contribute to poor clinical trajectories and developmental outcomes [5]. Anxiety has been associated with depressive symptoms and self-injurious behavior in children and adolescents with ASD [6].

Physical exercise may be a useful strategy to reduce anxiety and physical fitness in children with ASD. It is well-recognized that physical activity can have anxiolytic properties for individuals with mood disorders [7,8], with several mechanisms of action, including increased endorphins, alleviation of the physical symptoms of stress, improved self-confidence, and distraction. In this study, we focus on underserved children with ASD, although we assume that all children with ASD may benefit. Recent research has shown that physical fitness and obesity are a concern in children and adolescents with ASD, which may have negative long-term health consequences [9]. Exercise programs may improve physical health and fitness in addition to reducing anxiety [[10], [11], [12]]. Physical activity can also be helpful in improving emotion regulation and behavior functioning [13], stereotyped behavior [14,15], motor skills [16], social skills [16], cognition [17], and neuroinflammation [11] for individuals with ASD.

LEGO and Minecraft were utilized as an alternate activity comparison. While LEGO and Minecraft are known to bring about improvements to ASD symptoms [[18], [19], [20], [21]], there is minimal data confirming their efficacy in decreasing child stress. Since children with ASD are naturally drawn to repetitive, predictable, and repeating patterns, LEGO is an intrinsically motivating toy due to its highly structured, foreseeable, and systematic properties [[18], [19], [20]]. Minecraft is a type of electronic video game for psychotherapy (EGP), which are games that are specifically designed for therapeutic purposes [22]. EGPs are developed to strengthen psycho-education, attitude change, relaxation, social skills, problem-solving skills, self-control skills, motivation, and therapist-client interaction [21]. The sedentary LEGO and Minecraft activities were selected as comparison activities that were conducted at the same time and location of the physical exercise group.

There are critical gaps in knowledge regarding the feasibility and effectiveness of physical exercise and sedentary play-based therapies as an intervention to reduce anxiety in children with ASD. Prior to this study, we conducted a pilot phase of the study to gage response, feasibility, and safety of our basic concept. However, no prior randomized controlled studies have directly examined the feasibility and effect of structured physical exercise, specifically designed for ASD, or play therapy interventions to reduce anxiety in young children with ASD from underserved, low-income families. Our research aimed to close this gap in knowledge by gaining new insights into mechanisms about the effects of physical exercise and play therapy on anxiety in a group of children with ASD from underserved families. Recruitment of families from low income and medically underserved backgrounds was important, as treatment disparities exist [[23], [24]] and development of a low cost and easily replicable intervention can be especially impactful for those with fewer resources.

Participants engaged in a randomized 8-week physical exercise or sedentary intervention, with a primary outcome measure of Child Behavior Checklist DSM-5 (CBCL DSM-5) anxiety scores and secondary measures of salivary cortisol, the Screen for Childhood Anxiety Related Emotional Disorder (SCARED), the Child's Sleep Habits Questionnaire (CSHQ; Autism Treatment Network abbreviated version), and Physical Activity Questionnaire for Older Children (PAQ-C). We studied the effects of physical exercise or sedentary intervention on anxiety at the primary timepoint week 8, as well as weeks 3 and 6, and at extended follow-up 8 weeks after the end of the intervention program.

### Method validation

The results of this study are described in “The Effects of a Physical Exercise Program, LEGO and Minecraft Activities on Anxiety in Underserved Children with Autism Spectrum Disorder” [25]. Anxiety scores, as measured by the parent-rated CBCL DSM-5 anxiety subscale and the self-rated SCARED, showed improvements at weeks 3, 6, and 8 in both physical exercise and sedentary play groups, with no significant differences between groups. CSHQ-ATN sleep scores improved in the physical exercise group during weeks 6 and 8, but not in the sedentary play group with no significant difference between LEGO activity and Minecraft activity subgroups. Salivary cortisol did not show any significant changes from baseline to post-intervention in either group. The PAQ-C scores showed that physical activity level was increased in the physical exercise group compared to the sedentary play group at week 8.

## Research methodology

### Participant selection

Three hundred and eighty-two children with a current diagnosis of ASD were screened for participation in the study. The study was conducted in person at The Center for Autism and Neurodevelopmental Disorders, at the University of California, Irvine (UCI) in Santa Ana, CA and the Thompson Center (TC) for Autism and Neurodevelopmental Disorder at the University of Missouri in Columbia, MO.

Eligibility criteria for families included low income, autism diagnosis and high level of stress (evaluated using the CBCL DSM-5 anxiety subscale or SCARED). Families had to be covered by CalOptima and/or meet “low-income” guidelines as defined by the low-income category of HUD's Fiscal Year 2018 Income Limits. As part of the screening process, ASD diagnosis was confirmed using the Autism Diagnostic Observation Schedule-2 (ADOS-2). The ADOS-2 is a gold standard instrument used in ASD diagnosis and research. It is available in 4 different modules, with each module appropriate for different levels of language. Research staff reviewed children's medical records for ADOS-2 assessment within the past three years to ensure ASD scoring criteria. If there was no assessment on hand, the ADOS-2 was administered by approved research staff. The CBCL DSM-5 was used to evaluate level of stress and administered to all parents. SCARED was administered to children aged 8–18. The questionnaires were scored, and children who scored ≥ 93rd percentile on the CBCL DSM-5 anxiety subscale or a total score of ≥ 25 on the SCARED were eligible to participate.

The study PI or clinician further reviewed and screened any parent-reported information on the child's medication use, birth defects, genetic or medical conditions that may preclude participation or pose a risk during the study sessions. Additional inclusion and exclusion criteria listed below were developed to ensure participant safety and decrease the amount of confounding variables with regards to participant exercise activities. Our sample included children who participated in regular physical exercise at school or unstructured physical activity in the community or family. Only children who were actively participating in community-based exercise programs or who planned to begin an exercise program were excluded. This was to minimize confounding effects of extensive exercise programs, while also ensuring that children were able to continue their day-to-day routine. Inclusion and exclusion criteria are listed below.

#### Inclusion criteria

• Child has an ASD diagnosis as confirmed by ADOS-2.
• Child is 6 to 12 years old.
• Child has anxiety, scoring ≥ 93rd percentile on the CBCL DSM-5 anxiety subscale or a total score of ≥ 25 on the SCARED.
• Child and parent/caregiver are able to attend study sessions 3 times a week for 8 consecutive weeks from the consent appointment.
• Child is able to follow instructions.
• Child is able to participate in moderate physical exercise.
• Family meets underserved status: is either covered by CalOptima or Medicaid Insurance, and/or his or her family meets federal guidelines for low-income (as defined by the low (80%) income category of HUD's Fiscal Year 2018 Income Limits Summary for their respective area).

#### Exclusion criteria

• Child is a danger to themselves or others.
• Has a medical condition that may pose a risk during physical exercise.
• Child has joined a regular exercise program in the last 4 weeks up to the consent appointment.
• Child is currently in an exercise program and expected to discontinue it in the next 8 weeks from the consent appointment.
• Child is planning to join a new exercise program in the next 8 weeks after the consent appointment.
• Child has visual, auditory or motor impairments, which would preclude participation in study activities.

#### Sample size

Although there is little known about the effects of physical exercise interventions on anxiety in children with ASD, previous research has suggested that the effect sizes of physical exercise on other behavior is, on average, moderate to strong [26]. We intended to recruit 220 participants; allowing for a slightly higher dropout rate (20%) than observed in our previously conducted pilot study, we would have approximately 176 children with an 8-week measurement. This sample size would provide 90% power (using alpha = 0.05, two-sided) for a 2.5 point difference in the CBCL DSM-5 ratings in response to physical exercise compared to control group, allowing for a standard deviation (SD) of 5. This is approximately 1/3 of the effect previously reported in other studies [27,28] so we would have had more than adequate power for this endpoint. Sample size for the study was also deemed adequate to assess salivary cortisol endpoint. Previous research [10] showed a moderate effects size (partial eta square = 0.48) and an average level of 0.11 microgram/deciliter (SD = 0.05) before the first physical exercise session and 0.09 (SD = 0.04) microgram/deciliter after the last session. Assuming a correlation of 0.50 between measurements, 82 participants per group (*N* = 164) would be needed to provide 80% power to detect the 0.02 difference (assuming no change in the control group) (alpha = 0.05, two-sided).

However, we were unable to achieve the number of recruited individuals due to the 8-week time commitment and long-distance travel to the Missouri site. We recruited 148 subjects, of which 117 completed the study. Our drop-out rate for 8-week measurement was 21%, similar to the expected drop-out rate.

### Participant randomization

Participants were randomized according to a computer-generated randomization schedule managed by the AIR-P Data Coordinating Center (DCC) at the Massachusetts General Hospital (MGH) that assigned subjects equally (1:1) to the exercise and sedentary interventions, stratified by site. Participants assigned to the sedentary intervention were allowed to choose between LEGO or Minecraft.

Participants randomized to the sedentary intervention group were offered the option of crossing over to the exercise intervention immediately after completion of the initial placement. This is because our study group had hypothesized that the exercise intervention would benefit children more than the sedentary intervention group, and we wanted to ensure that all children had the opportunity to participate in the exercise intervention.

### Interventions

Participants engaged in their assigned intervention up to 3 times a week for 8 consecutive weeks. Each session lasted approximately 45 min and was led by a trained instructor (for physical exercise intervention) or trained researcher (for sedentary intervention). The duration and frequency of sessions was primarily informed by previous studies conducted on physical exercise interventions for individuals with ASD [26,10], as well as feasibility.

The duration of an 8-week intervention was chosen because many previous exercise interventions with individuals with ASD had a length of 8–36 weeks [14,10]. In a meta-analysis of physical exercise studies, it was discovered that 8 weeks is an adequate amount of time to have significant effects on health and behavior [29]. Physical exercise regimens were chosen to follow the exercise recommendations issued by the US Department of Health and Human Services. Children should exercise 60 min or more of moderate-to-vigorous intensity physical activity each day and include higher intensity aerobic exercise, muscle strengthening, and bone strengthening exercises at least three times per week [30]. Thus, we incorporated high intensity aerobic exercise, muscle strengthening, and bone strengthening into our protocol and encouraged participants to attend all three sessions.

Since the program is designed for children with ASD with a short attention span, we chose to limit the study sessions to 45 min, 3 times per week for 8 weeks for feasibility and economic reasons.

#### Exercise intervention

This eight-week program was administered in small groups and was designed to incorporate the key guidelines for physical exercise in children from the U.S. Department of Health and Human SErvices [30]. We adapted these guidelines to the needs of our population to be engaging and safe.

All exercise sessions were facilitated by trained instructors and structured in the following way:

1. 10-minute warm-up that included video modeling stretches and movements incorporating flexibility, muscle stretching and light aerobic exercise.
2. 10-minute aerobic exercises. Participants watched a 10-minute video model (with preferred music) consisting of exercises such as jumping, running in place, dancing movements, and side shuffles.
3. 3-minute water break.
4. 5-minute bone strengthening activity. This included teams engaging in tug of war games and wall push-ups.
5. 5 min of aerobic activity such as jumping in place or jumping on trampolines holding on to the bars.
6. 3-minute water break.
7. 5 min of aerobic/bone-strengthening activities such as a timed obstacle course that included running, jumping, and crawling.
8. 5 min of cool-down exercises including a 5-minute video modeling of stretches and movement incorporating flexibility, muscle stretching, and light aerobic exercise.

#### LEGO therapy procedures

The one-on-one LEGO therapy sessions consisted of one child building a LEGO set in collaboration with a student researcher. The child and the student researcher were assigned an “engineer” and “builder” role, respectively. The “engineer” gives verbal descriptions of the pieces needed and directions on how to assemble the model. The “builder” follows the directions, collects the pieces, and puts the pieces together. In LEGO therapy, the child switches between being a “builder” and an “engineer” so they can experience both roles. Another variation of LEGO therapy is demonstrated in ‘‘freestyle’’ LEGO activities, in which children design and build their very own model from scratch. This allows children to practice compromising, expressing ideas clearly, and taking other people's perspectives and ideas into account.

The protocol sequence is the following (for all participants):

1. For the first five minutes of the session, participants were introduced to their student researcher and participated in creating LEGO rules.
2. After the participants were assigned their roles, they played their role for approximately 15 min.
3. Once the first 15 min are over, the child switched roles and played for another 15 min. This division of labor with a common purpose allowed children to practice joint attention, turn taking, sharing, joint problem solving, listening, and general social communication skills.
4. For the final 15 min of the session, participants cleaned up the LEGO bricks, and answered a few questions on how they felt about the session (i.e., Was it hard or easy? What are your strengths and weaknesses? What do you want to work on?).

#### Minecraft therapy procedures

The one-on-one Minecraft therapy sessions consisted of one child building and exploring the Minecraft world with a student researcher with 2 computers and headsets. The child would be given goals and tasks for each specific session by the student researcher. They would collaborate to complete the tasks through verbal communication using headphone sets. This protocol allowed children to be creative, problem solve, and work towards a common goal with their student researcher.

The protocol sequence was the following (for all participants):

1. For the first 5 min of the session, participants were introduced to their student researcher and discussed their goal for that session.

a. Some examples of the goals for each session are making basic wood tools, crafting a bed, making an animal farm; all to achieve the goal of building a two-story shelter as a protection. This division of labor with a common purpose allows children to practice joint attention, turn taking, sharing, joint problem solving, listening, and general social communication skills.

2. After the participants were assigned their goals, they worked together to achieve the goal in the daytime mode for approximately 20 min.
3. Once the first 20 min are over, the game switched to night mode, where the participant and the student researcher had to survive the night with the products of the daytime mode for another 20 min.

a. During the night mode, the first 15 min are spent on surviving the night while the remaining 5 min are spent discussing how they felt about the session (i.e., Was it hard or easy? What are your strengths and weaknesses? What do you want to work on for the next session?).

### Outcome measures

Participants and their parents took questionnaires at specified times. These time points were chosen to assess progression throughout the intervention while also reducing the time commitment for parents. Schedules for the intervention and measures are shown in Table 1 below.

**Table 1:** Schedule of procedures and measures at study time points: all participants for first eight weeks.

		Week								
Procedure/Measure	Purpose	Screen	1	2	3	4	5	6	7	8
ADOS-2	ASD Diagnosis	X								
Intervention & attendance tracking			x3	x3	x3	x3	x3	x3	x3	x3


			Day 1		Day 1			Day 1		Day 3
CBCL DSM-5	Primary Anxiety Measure	X	X		X			X		X
PAQ-C	Physical Activity Measure		X		X			X		X
SCARED (aged 8–18)	Secondary Anxiety Measure	X	X		X			X		X
Salivary cortisol collection	Secondary Anxiety Measure		x3		x3			x3		x3
CSHQ-ATN	Secondary Sleep Measure		X		X			X		X

#### Primary outcome measures

The primary outcome of this study was the effect of the intervention on the parent-rated anxiety level, as measured by the CBCL DSM-5 anxiety subscale. The CBCL DSM-5 is a parent questionnaire that is part of the Achenbach System of Empirically Based Assessment, covering a wide age range. Raw scores from parent forms are converted to T-scores that describe functioning in DSM-oriented and Syndrome scales that assess problems with anxiety, somatic, social, internalizing, oppositional-defiant, conduct and aggressive behaviors, attention and hyperactivity. For this study, we focused on the anxiety subscale specifically.

Many studies that explored the effects of physical exercise on anxiety in children with other neurodevelopmental disorders utilize the parent form of the CBCL DSM-5 [[27], [28], [31], [32]]. The validity and reliability of the CBCL DSM-5 anxiety subscale is acceptable in children with ASD [33]. In addition to being a widely used measure, the CBCL DSM-5 is available in English and Spanish.

Parents completed the CBCL DSM-5 at their screening visit, four times during the intervention (Week 1, 3, 6, 8), and twice for extended follow-up (Week 12, 16). The Week 1 CBCL DSM-5 was completed during the first session of the week and was considered the baseline measure. Week 3 and 6 measurements were also completed during the first session of the week to measure progress through the intervention. The Week 8 measurement was completed during the last session of the week and was considered the post-intervention measurement. The week 12 and 16 measurements were completed to assess long-term impacts of the intervention. Data collection of the CBCL DSM-5 and all other surveys occurred via a web-based data entry system to allow easy access across different institutions.

#### Secondary outcome measures

Secondary measures included the SCARED, the CSHQ, and salivary cortisol which were measured at baseline, week 3, 6, and 8. Only week 1 and week 8 samples of salivary cortisol were assayed for the purpose of this study. Potential confounding measures included the PAQ-C, which was measured at baseline, weeks 3, 6, 8, 12, and 16.

The SCARED is a 41-item instrument to screen for childhood anxiety disorders in children aged 8 or older. It is a reliable and valid instrument utilized often in clinical settings [34], and has been used in children with ASD with acceptable validity, reliability, and sensitivity but low specificity [35,36]. Research staff explained all questions to 8–11-year-olds or those children who could not read the items. No SCARED questionnaires were excluded based on invalid responses.

The CSHQ-ATN (3-point scale) is a retrospective, 23-item parent questionnaire that examines sleep behavior in young children including key sleep domains associated with clinical sleep complaints of this age group Sleep problems are commonly associated with anxiety in children with ASD [37,38] and can also be benefited with exercise programs [[39], [40]]. Thus, studying sleep habits provided valuable insight into alleviation of anxiety symptoms for children participating in the interventions. The CSHQ-ATN was specifically modified to better assess sleep problems in children with ASD [41], and has been validated and widely utilized to assess sleep problems for children with autism [[42], [43], [44]]. The CSHQ is also available and validated in both English and Spanish [45].

Salivary cortisol is a well-recognized independent stress biomarker [[46], [47]] with moderate reliability as a recovery marker in physical exercise [48]. Salivary cortisol collection occurred before intervention, immediately after, and 20 min after intervention. These time points were chosen as sensitive time points for identifying acute stressors such as physical exercise [49,50], and have been previously utilized to assess cortisol measurements for young adults with ASD [10]. All interventions and measurements took place between 4:30 – 6:00 pm. This collection time was chosen to accommodate participant schedules, but also represents a preferential time for cortisol collection since standardized collection of salivary cortisol in the late afternoon has less variability between the participants compared to collection in the morning [51,52].

For the salivary cortisol collection, participants were instructed not to eat or drink for at least 30 min before arriving for the intervention session. Upon arrival, participants rinsed their mouth with water and rested for 10-minutes. Saliva was collected using Salimetrics Children Swab® (Salimetrics LLC, State College, PA) or by the child directly spitting into the specimen collection tube. When using the swab, the swab was placed under the tongue with the help of a research assistant for approximately 2 min to saturate the swab with saliva, and then placed into a swab storage tube and stored immediately in the −20 °C freezer. All samples were transferred weekly to be stored at −80 °C until assayed. The swabs were thawed at room temperature in small batches, saliva was extracted using centrifugation at 1500 g for 15 min. Extracted saliva was stored in aliquots at −80 °C until cortisol assay. For the cortisol assay, saliva cortisol concentrations were determined using a high sensitivity salivary cortisol enzyme immunoassay kit with a detection range from 0.012 to 3 µg/dL according to the manufacturer's instructions (Salimetrics, State College, PA; Item No. 1–3002). All samples were run in duplicates along with standards and controls. Absorbance was measured spectrophotometrically at 450 nm using a microplate reader (Molecular Devices spectramax Pro 384) with background correction at 550 nm to control for plate imperfections. The cortisol concentrations in the saliva samples were predicted based on the standard curve generated using a four-parameter logistic (4-PL) curve fit using Softmax Pro (Molecular Devices) software.

The PAQ-C is a parent report instrument that provides reliable and valid information on general physical activity for the week prior to administration [53,54]. This was utilized to measure physical activity that children engaged in outside of the intervention ranging from moderate to vigorous activity. The PAQ-C has been widely used to assess physical activity participation in children aged 8–18 with ASD [[55], [56], [57], [58], [59]].

### Study progression

As seen in the graphical abstract, most participants participated for 16 weeks, with an 8-week (physical exercise or sedentary play only) intervention and 8-week follow-up. Some participants had a 24-week study, with a 16-week (sedentary and cross-over physical exercise) intervention and 8-week follow-up.

#### Orientation

Each participant was asked to undergo one session to assess eligibility, explain the purpose of the study and to obtain informed consent. The screening session was held privately among the researchers, the participant, and the participant's parents or legal guardians. During the screening session, participants completed the ADOS-2 (if required), CBCL DSM-5, and SCARED (if applicable) surveys to fulfill screening criteria. Participants and parents or legal guardians also learned more about the study procedures such as the strategies utilized in LEGO and Minecraft therapy and the role of research personnel. All questions from participants and parents or legal guardians were answered, and informed consent forms were signed during this screening session as well.

The participants and their parents were also introduced to the space and measurements before starting the study. Study staff who were trained in behavior therapy and applied behavior analysis helped participants who needed extra accommodations. Children who had difficulty adjusting were given time to observe and incrementally rewarded for participation in the study procedure.

#### Extended follow-up

Participants completed the CBCL DSM-5 and PAC—C questionnaires at timepoints 12 and 16 (4 weeks and 8 weeks after their final intervention session respectively) to assess any long-lasting effects of intervention on anxiety and exercise habits, respectively. No cross-over participants were included in extended follow-up analysis to avoid confounding results, since they participated in both interventions. These time points are commonly used in other post-intervention follow up studies.

#### Cross-over participants

Cross-over participants were allowed to cross over to the physical activity intervention immediately after they completed the 8 weeks of sedentary intervention. For the cross-over period, they followed the same schedule shown in Table 1 above. While this minimized the disruption to participant's schedules and allowed them to complete both interventions on a shorter time scale, it did result in a loss of week 12 and week 16 follow up for the sedentary intervention in these participants.

### Data analysis

A repeated measures ANOVA with fixed effects for baseline value of the given outcome, treatment group, post-baseline timepoint (week 3, week 6, and week 8) and an interaction between time point and treatment group was used for each primary and secondary outcome measure (see [25] for more details).

## Complications and additional considerations

### Subject attendance

Participants were encouraged to attend all 3 weekly sessions. To encourage attendance, we provided monetary incentives for completion of the study. In addition, we made efforts to accommodate school schedules and work schedules of caregivers. For example, late afternoon sessions were preferred by participating families, hence both exercise and sedentary interventions were administered between 4:30 – 6:00 pm. We also attempted to optimize the experience for intervention for the individual and improve retention by documenting in-session compliance and enjoyment during each session for both groups. This documentation was used internally to adjust future sessions.

**Fig. 1:**
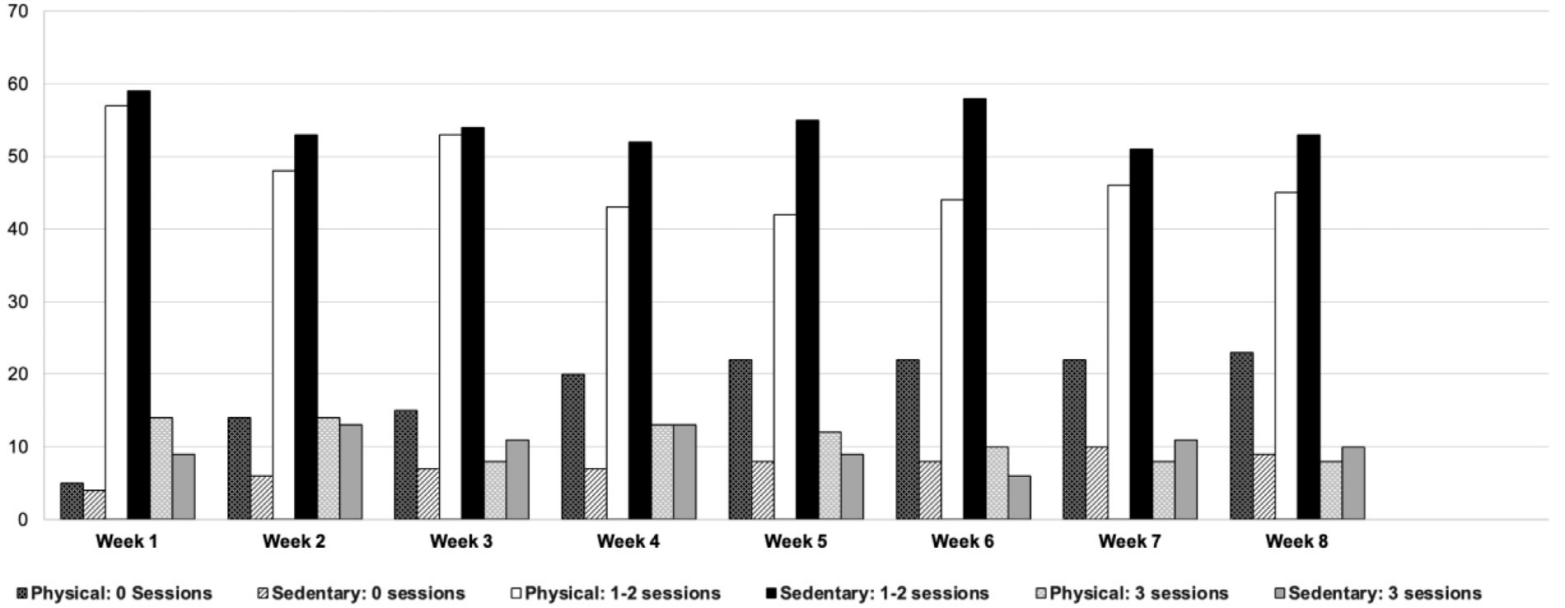
Participant Attendance for 8 weeks of Intervention.

However, most participants were unable to attend 3 times weekly, and instead attended 1–2 sessions per week. Factors which influence participation included ease of access, e.g., transportation and programming that does not negatively affect caregiver work schedules. Participation attendance was documented in Fig. 1 below.

To adjust for this variability in attendance, session attendance was monitored and documented for all participants throughout the course of the study. If the participant missed a week due to an illness or other unforeseen events, they had a chance to make up that week. Participants were discontinued from the study if there were more than four weeks of consecutive absences, or if there were more than six weeks of absences total. Acceptable compliance with the program is defined as participation at least once per week for the eight weeks of session participation.

### Working with underserved families

As mentioned in the Study Background, the study aimed to assess the feasibility and efficacy of tailored physical exercise programs to reduce anxiety in children with ASD from underserved backgrounds, especially Latino and rural families. As such, all test sites were prepared to recruit both English- and Spanish-speaking families, and parents were permitted to choose between English or Spanish versions of the surveys. 34% of our participants were Spanish speakers and utilized the Spanish version of the CBCL DSM-5 and CSHQ-ATN.

### Monitoring adverse effects (AEs)

Participants were monitored for adverse effects from the time they signed consent until they completed the last study visit. Families were asked to call the site Principal Investigators or research staff if they experience discomforts after participation. All families were provided with a 24-hour phone number to call in the event of clinical need.

No adverse effects were encountered during the investigation. While some participants struggled and displayed disruptive behavior during the intervention, trained research assistants were able to aid the participants without requiring intervention of principal investigators.

### Potential adaptations of this methodology

Adaptations of this methodology may also have use in studying the effects of physical activity or play therapy interventions on depression and other mental health disorders, individuals with ASD or other neurodevelopmental disorders, or testing different types of intervention.

For future studies, community-based settings such as school or after-school programs could ease the burden of attendance and improve ability to explore dose as a variable. In addition, tele-exercise interventions conducted in the home environment may be an alternative for families who live in remote areas, lack transportation, or want to maintain social distance during a pandemic.

## Ethics statements

The study was IRB approved and informed consent was obtained from subjects.

## CRediT authorship contribution statement

**Angelina Lo:** Writing – original draft, Visualization. **Lea Ann Lowery:** Methodology, Conceptualization, Investigation. **Karen Kuhlthau:** Methodology, Conceptualization, Investigation, Writing – review & editing. **Robert A. Parker:** Formal analysis, Writing – review & editing. **James Chan:** Formal analysis, Writing – review & editing. **Fadia Haddad:** Methodology, Conceptualization, Investigation. **Shlomit Radom-Aizik:** Methodology, Conceptualization, Investigation, Writing – review & editing. **Jean-G. Gehricke:** Methodology, Conceptualization, Investigation, Writing – review & editing, Supervision, Project administration, Funding acquisition.

## Declaration of Competing Interest

The authors declare that they have no known competing financial interests or personal relationships that could have appeared to influence the work reported in this paper.

## Data Availability

Data will be made available on request.
